# *“You are not the person you used to be…”.* A phenomenological study on sexual desire and embodied sexuality in heterosexual women with fibromyalgia syndrome

**DOI:** 10.1080/17482631.2026.2732815

**Published:** 2026-09-23

**Authors:** Patricia Romero-Alcalá, José Granero-Molina, María Rosario Coín Pérez-Carrasco, Anabel Chica-Pérez, Carmen Ramos-Rodríguez, María del Mar Jiménez-Lasserrotte

**Affiliations:** a Faculty of Health Sciences, University of Almería, Almería, Spain; b Nursing, Physiotherapy and Medicine Department, University of Almería, Almería, Spain; c Faculty of Health Sciences, Universidad Autónoma de Chile, Santiago, Chile; d Clinical Pharmacy, Andalusian Health System, Málaga, Spain; e Fibromyalgia Association of Almería, Almería, Spain

**Keywords:** Fibromyalgia, sexual desire, sexuality, partner, qualitative study

## Abstract

**Purpose:**

The objective of this study was to describe and understand how women with fibromyalgia syndrome (FMS) perceive their sexuality and the changes associated with sexual desire.

**Method:**

A qualitative methodology based on Merleau-Ponty’s phenomenology was used. Data collection included in-depth interviews with fifteen women diagnosed with FMS.

**Results:**

Two main themes emerged from the results: “When desire moves between light and shadow” and “Only your partner can understand the traffic light of desire”.

**Conclusions:**

Sexual desire is substantially affected by FMS. Understanding the fluctuations in desire and how both the multiple symptoms of FMS and the relationship with one’s partner influence it may help individuals adapt to their new emotional situation and sex life.

## Introduction

Fibromyalgia syndrome (FMS) is a multisystemic rheumatic syndrome characterised by widespread chronic musculoskeletal pain as well as emotional, cognitive, and sleep disorders (Ablin et al., 2011; Wolfe et al., 2016). FMS is a public health problem, with a global prevalence ranging from 2–5%, specifically 2.3% in the European population and 2.4% in the Spanish population (Cuevas-Toro et al., 2025; Kuculmez, 2025). FMS is more prevalent among women, with rates ranging from 3.6% to 5.6%, increasing with age (Kocyigit & Akyol, 2022). FMS is characterised by the heterogeneity of symptoms (Ablin & Wolfe, 2017), primarily chronic widespread musculoskeletal pain and pressure in various parts of the body (Kuculmez, 2025), sleep disorders (de Souza et al., 2025), stiffness upon waking (Martín-Pérez et al., 2025), fatigue (Aldarwesh, 2025), emotional stress (Ghiggia et al., 2025), anxiety (Galli et al., 2025), depression (Sedda et al., 2025), and cognitive impairment (Abdulazim et al., 2024).

FMS is associated with female sexual dysfunction (FSD) (Besiroglu & Dursun, 2019; Flegge et al., 2023; Kayhan et al., 2016; Ricoy-Cano et al., 2022), characterised by pain, difficulty reaching orgasm, lack of sexual satisfaction, and lack of desire (Mollà-Casanova et al., 2023; Ricoy-Cano et al., 2022). It also leads to a decline in self-esteem, intimate relationships, sexual health, and quality of life for women (Cuevas-Toro et al., 2025; Demir et al., 2025). FMS management should include a combination of pharmacological and non-pharmacological treatments; a personalised treatment which takes into account the individual characteristics of each woman (Kocyigit & Akyol, 2022). No specific treatment exists for FMS, but rather a multidisciplinary approach focused on reducing pain and symptoms (Filipovic et al., 2025; Giorgi et al., 2023), physiotherapy (Albuquerque et al., 2022), and therapy for emotional and psychosomatic difficulties (Antonelli et al., 2025). Non-pharmacological interventions are recommended as the first line of treatment including aerobic exercise, cognitive-behavioural therapy, and patient education, all of which are aimed at alleviating pain and other symptoms. Additional approaches have been studied, such as digital health interventions, combination therapies, and non-invasive neuromodulation, among others. Pharmacological therapy aims to promote pain modulation (Martínez & Guimarães, 2024). FDA-approved drugs such as duloxetine, milnacipran, and pregabalin have demonstrated a reduction in moderate-to-severe pain intensity in at least 50% of affected individuals (Moore et al., 2025). Recent studies recommend holistic treatments (Triantafyllias et al., 2025); however analgesics, corticosteroids (Giorgi et al., 2023), or anxiolytics and antidepressants continue to be used, with negative side effects on sexual response due to reduced vaginal lubrication (Mollà-Casanova et al., 2023), lower sexual satisfaction (Matarín-Jiménez et al., 2017; Van Overmeire et al., 2022), and lack of desire (López-Rodríguez et al., 2019; Ricoy-Cano et al., 2022).

Sexual desire is a complex and multidimensional term (Ågmo, 2025; Chesli et al., 2024). It is defined as the subjective psychological state of initiating and maintaining sexual behaviour, and can be triggered by internal stimuli (feelings, thoughts, erotic fantasies) and/or external stimuli (partners, situations, pornography) (Nimbi et al., 2024; Vowels et al., 2021). Desire is a key component of sexual response (Chesli et al., 2024). While solitary desire increases in the absence of a partner (Wieczorek et al., 2022), dyadic desire is associated with perceived fairness, romantic feelings, and sexual satisfaction (Blumenstock et al., 2024; Johansen et al., 2023; Vowels et al., 2021). Marital adjustment is key to adapting to FMS (Granero-Molina et al., 2016; Pereira et al., 2025), where desire, sexuality, and the couple’s relationship is strongly affected (Pereira et al., 2025; Romero-Alcalá et al., 2019). Several studies explore the relationship between FMS and FSD (Besiroglu & Dursun, 2019; Deggerone et al., 2024; Mollà-Casanova et al., 2023; Ricoy-Cano et al., 2022), medication effects (Cuevas-Toro et al., 2025; López-Rodríguez et al., 2019; Van Overmeire et al., 2022), or orgasm disorders/female sexual satisfaction (Ablin et al., 2011; Mollà-Casanova et al., 2023). However, few studies examine how women with FMS experience their sexuality (Demir et al., 2025; Granero-Molina et al., 2016, 2023; Matarín-Jiménez et al., 2017), or the experiences of their partners (Romero-Alcalá et al., 2019). To our knowledge, few qualitative studies have specifically focused on the lived experiences of sexual desire among women with FMS (Santos-Iglesias et al., 2022). According to Merleau-Ponty, our body is our way of relating to the world while sexuality is a way of being and relating to others as embodied beings (Glenn, 2022).

### Objective

The objective of this study was to describe and understand how women diagnosed with FMS perceive their sexuality and the changes associated with sexual desire.

## Materials and method

### Design

A qualitative study that uses Merleau-Ponty's (MP) phenomenology was developed (Merleau-Ponty, 2013). Theories of the body and perception are closely linked in Merleau-Ponty's philosophy; our body is our way of relating to the world, distinguishing between the objective body and the “lived body”. The condition (FMS) reveals the bodily nature of being, how the body experiences, reacts, and manifests; along with each person's understanding of the world, making it relevant to our study. Merleau-Ponty examines immediate experiences. According to MP, sexuality is a way of being in the world and a way of relating to others as embodied beings. It is this experience of embodied subjectivity that allows us to understand the consciousness of the other through language (Glenn, 2022). Through phenomenological reduction, our consciousness is freed from pre-existing categories and allows us to explore the experiences of sexual desire in women with FMS. This study explores the phenomenon of sexual desire in women with FMS in the ways in which the women themselves embody it (Granero-Molina, 2019). The Consolidated Criteria for Reporting Qualitative Research (COREQ) criteria were applied when writing the manuscript (Tong et al., 2007).

### Participants and setting

The participants were women diagnosed with FMS. Recruitment was facilitated by primary healthcare professionals and two FMS patient associations, who informed the women about the study and requested their participation. The sample was obtained from a population in three different cities in eastern and southern Spain. The inclusion criteria required participants to be women of legal age, diagnosed with FMS for ≥2 years, and willing to provide informed consent. Women diagnosed with rheumatic or psychiatric conditions or receiving psychological therapy, were excluded from the study. A total of 25 participants were selected using purposive sampling. Five declined to participate because they did not want to discuss their sexuality, three because they did not have time, and two because they believed their partner would not approve. Before the study began, participants received an explanation of the objective and ethical considerations related to the research. All interviews were audio-recorded with the prior written consent of the participants. The final sample consisted of 15 participants (Table I), with a mean age of 54.4 years, SD = 13.2, and a mean of 13.2 years since receiving the FMS diagnosis. All participants identified as female and heterosexual, 73.3% are married women, 13.3% live with an unmarried partner, one is single, and another is separated with no partner.

**Table I.** Sociodemographic data of the participants (*N* = 15).

Participants	Sexual orientation	Profession	Marital status	Years with FMS
IDI1	Heterosexual	Self-employed	Married	18
IDI2	Heterosexual	Retired	Single	16
IDI3	Heterosexual	Waitress	Married	9
IDI4	Heterosexual	Self-employed	Married	20
IDI5	Heterosexual	Shop assistant	Married	7
IDI6	Heterosexual	Pensioner	Married	8
IDI7	Heterosexual	Retired	Married	5
IDI8	Heterosexual	Pensioner	Married	10
IDI9	Heterosexual	Self-employed	Separated	8
IDI10	Heterosexual	Retired	Married	40
IDI11	Heterosexual	Student	Unmarried partner	5
IDI12	Heterosexual	Retired	Married	11
IDI13	Heterosexual	Retired	Married	21
IDI14	Heterosexual	Retired	Married	15
IDI15	Heterosexual	Caregiver	Unmarried partner	5

IDI = In-Depth Interview; FMS = Fibromyalgia Syndrome.

### Data collection

Fifteen in-depth interviews (IDIs), conducted individually and privately in the Spanish language, were carried out between February and May 2025. Limited access to participants necessitated conducting six interviews online or by telephone. The interviews had an average duration of 41 minutes. The in-person interviews took place in a quiet, private room at an FMS patient association, with only the participant and researcher present. The aim was to explore the lived experiences of women with FMS, mediated by their backgrounds and contexts. Following the development of a well-structured phenomenological interview protocol, interviews with open-ended questions were conducted by the principal researcher, who had not previously met the women (Table II). Each participant completed a single recorded individual interview, and notes were compiled along with a reflective journal for the description of the results. Bracketing techniques allowed researchers to become aware of and manage their assumptions regarding the phenomenon. Data collection was stopped once data saturation was reached (no new units of meaning emerged that would modify the interpretation of the data).

**Table II.** Interview guide.

Stage of the interview	Theme	Content/example question
Presentation	Motives Intentions	Belief that their experience should be well-known to all Carry out research to raise awareness of their experience
Start	Starting questions	Could you tell me about your experience of sexual desire with FMS?
Development	Guide for the conversation	Describe, based on your experience, the causes of the loss of your overall sexual desire Can you describe the moment when you first experienced a lack of sexual desire? What does the relationship between your medication for FMS and sexual desire suggest to you? Could you tell me more about how you feel that antidepressants, analgesics, or anxiolytics affect your sexual desire? How do sleep problems, fatigue, and tiredness affect your individual and partnered sexual desire?
Close	Final question Thanks	Is there anything else you’d like to add? We thank you for your time and are available if you need anything

FMS: Fibromyalgia Syndrome.

### Data analysis

All of the interview recordings and notes were transcribed, incorporated into a hermeneutic unit, and analysed using ATLAS.Ti25 software. Relating Merleau-Ponty's philosophy of embodiment to Georgi's method (Giorgi et al., 2017) entails understanding how the theory of the “lived body” is operationalised into a data analysis tool. Data analysis was conducted by PRA, JGM, and MMJL, who discussed differences until reaching a consensus. The results were reviewed by two independent researchers, but the structure of the findings remained unchanged. The data analysis (Table III) follows five steps (Englander & Morley, 2023). **Step 1.** Initial reading to obtain a sense of the whole: researchers read the complete transcripts of the participants' phenomenological interviews to gain a global sense of the narrative and understand the “climate” of the lived experience. **Step 2.** Adopting the phenomenological psychological attitude: *epoché* invites researchers to set aside their own preconceptions, to describe their pre-analytical intuitions and to make notes (memos) in ATLAS.ti. The process carried out by researchers throughout the analysis involves: articulating hypotheses/experiences (pre-interview); refraining from advising, suggesting, or steering the discourse (during the interview); and verbatim transcription and bias mapping (during the analysis). **Step 3.** Dividing data into units of meaning: the researcher breaks down the transcripts into smaller parts (units of meaning) to allow for a more detailed and closer analytical approach to the data. **Step 4.** Transformation of everyday expressions to psychological meaning: researchers extract the most complete meanings embedded within the participants’ descriptions, an inductive process in which the first ideas regarding subthemes and themes emerge. **Step 5.** Returning to the whole and moving toward the general structure: synthesising the parts into a structural narrative. Three researchers extracted definitive subthemes and themes. Discrepancies were discussed with research team members until a consensus was reached.

**Table III.** Example of data analysis.

Quote	Initial code	Units of meaning	Subtheme	Teme
“The least helpful thing is when they ignore you, they don’t notice how you feel or understand what you’re going through, […] When they tell you that you’re exaggerating your symptoms… you stop thinking positively about everything, especially sex.” (IDI15)	stress, anxiety, pain, lubrication, lack of desire, lack of interest, rushing	Feeling understood, making time for ourselves, taking it slow	When the desire killers arrive	Desire moves between light and shadow

### Ethical issues

The study was conducted in accordance with the ethical principles of the Declaration of Helsinki. Approval was obtained from the Ethics and Research Committee of the Department of Nursing, Physiotherapy and Medicine at the University of Almería (N˚ 22/2017). The participants were informed of the study’s objective, the voluntary nature of their participation, and the possibility of withdrawing from the study at any time. Anonymity was guaranteed in accordance with Organic Law 3/2018 on the Protection of Personal Data and Guarantee of Digital Rights. The authors report no competing interests to declare.

### Rigour

This study followed the quality criteria proposed by Lincoln and Guba (1985). Credibility was strengthened through prolonged engagement with the data, iterative analysis, and ongoing reflexive engagement throughout data collection, coding, and theme development. The data collection process was detailed; the main researcher was experienced in conducting in-depth interviews and encouraged participants to support their statements with examples. Transferability: a detailed description of the setting, study context, method, and participants is provided, enabling readers to assess the applicability of the results to other contexts. Dependability: two experts, independent of the data collection and analysis, read the transcripts, identified codes separately, and discussed differences in their interpretation of the data. Verbatim participant quotations were incorporated to enhance transparency of the findings. Confirmability: an audit log was maintained documenting the research process, decision-making, and the drawing of conclusions from the data.

### Reflexivity

Our reflective practice did not aim to eliminate pre-understanding, but rather to raise awareness of and manage the researchers' assumptions. A literature review was conducted following data collection, and the participants' descriptions of their experiences regarding desire were examined, while avoiding interference from the team's other research on female sexuality in FMS. This was a complex process for the first author, a psychologist and sexologist pursuing doctoral studies in this field. Data analysis sought to distance itself as much as possible from physiological descriptions, focusing instead on the body-subject duality. Power dynamics were analysed, and the study's paradigmatic orientation was discussed. Although Merleau-Ponty’s descriptive phenomenology was adopted, this complicated procedures such as the *epoché.* Reflection also focused on how the recruitment of patients from primary health care settings, some of whom had other medical conditions, might have influenced the results. The perspective of a clinical psychologist, president of the FMS Patients Association, influences the interview guide, monitoring, and interpreting the data. The other researchers do not engage in clinical practice, but have experience in qualitative research on FMS, while also examining their preconceptions. No differences were observed between in-person and online interviews, although the choice of a telephone conversation by many participants was surprising. This resulted in a loss of information regarding gestures or body language during the interview. To mitigate bias and leverage collective experience, the first author kept a reflexive journal and recorded voice notes, which were reviewed and discussed in team meetings.

## Results

The data analysis reveals two themes and six subthemes that allow us to describe how women with FMS perceive their sexuality and the changes associated with desire (Table IV).

**Table IV.** Themes and subthemes.

Theme	Subtheme	Units of meaning
1. When desire moves between light and shadow	1.1 My desire left one day and never returned	Desire and FMS: is it age or FMS? Self-esteem and sexual relations: where does sex fit in now? Changes in sexual encounters
	1.2 I'm having a good day today	Mood and desire, going all in, disconnecting to reconnect, planning dates, having sex, relieving stress, improving sleep
	1.3 When the desire killers arrive	Without sleep, there is no sexual desire, I am not exaggerating, explicit stimulation does not work, stress can be a lethal companion, pain takes your desire away, medication, desire, and lubrication
2. Only your partner can understand the traffic light of desire	2.1 If you’d look at me…	Understanding, feeling seen, feeling like a woman, patience is the greatest remedy, seeing each other as we used to
	2.2 When things don’t flow smoothly…	Fear of not meeting expectations, maybe it is better to just stop, I understand myself better on my own, painful orgasms, healthcare professionals do not provide answers
	2.3 FMS belongs to both of us	Communication, gradual understanding, adjusting the intensity, facing it together, setting aside time to be together, games, stress relief

FMS: Fibromyalgia Syndrome.

### Theme 1. Desire moves between light and shadow

Sexual desire in women with FMS can be negatively affected by multiple physical and psychological factors. Medication use, hormonal imbalances, and diminished self-esteem can shift sexuality away from being a priority, thereby reducing the frequency of sexual encounters. Each woman attributes these changes to various personal factors and circumstances, coping with them as best as she can. Some women seek help from specialists but do not receive answers; thus, they enjoy the days when their sexual desire is strong, and avoid sexual relations when it is absent.

#### My desire left one day and never returned

Most women associate sexual desire and FMS as antagonistic concepts. When they are experiencing an FMS flare-up, sexual desire disappears.

*“They’re two completely different things that don’t go together. When you’re having an FMS flare-up, sex is the last thing on your mind […] When you’re feeling that unwell, it’s hard to have sex because your body can’t handle it. It’s impossible! They go hand in hand: when you’re feeling better, you’re in a better mood, you want to get ready and look nice…”* (IDI1)

Other women do not directly relate FMS to a lack of sexual desire, but agree that when they are in pain, they feel no desire. While pain flares are described in different ways, they always negatively impact sexual desire. As some participants described it:

*“I associate it with: I don’t feel like it right now, this hurts, that hurts, my kidneys hurt today, my knee hurts today. I’m not in the mood. I’m never in the mood. So of course, your sex life suffers because if you’re in pain it affects your mood and being in a bad mood affects your sex life”* (IDI10)

*“In my case, I don’t think I have any problem with it […] As long as I’m not going through a pain flare-up.”* (IDI2)

Some participants, after researching and speaking with specialists, associate their lack of sexual desire with hormonal changes, having more to do with menopause than FMS.

*“I think it also has to do with my age. I’m 47 […], I can’t blame everything on FMS. […] I talked to my primary care doctor about it because I’ve always been very sexually active, […] but there came a point, two or three years ago, when my libido just disappeared and never came back. I’ve been to therapy, seen a sex therapist, talked to my doctor, my gynaecologist, my internist…I’ve talked to everyone because I want my libido back, but I haven’t had any luck.”* (IDI4)

Another factor that affects many participants is self-esteem, as the women do not think of their bodies as something to take care of, enhance, or display. A positive body image contributes to improved well-being, feeling attractive, and being more open to sexual encounters.

*“Not just as a woman, but as a person, you lose some of your self-esteem and self-love, the way that you love yourself changes. You stop taking care of yourself physically […] Today I’m ok, tomorrow I’m not. You stop getting ready and you get stuck in a cycle of sweats, a ponytail, laying on the sofa under a blanket. As a woman, you don’t feel very feminine, you don’t love yourself, you don’t take care of yourself… it negatively affects your relationship.”* (IDI5)

Sexual relationships become a secondary aspect of participants’ lives. In general, rather than describing a transformation, women generally point to a reduction in sexual desire attributed to age, hormonal changes, or FMS. Not only does the frequency decline, but also the manner and intensity of sexual encounters.

*“I used to be very sexually active, extremely […] But I’ve had to tone it down; it can’t be that rough anymore, everything has to be gentler, more relaxed. I have to be more careful with positions; my arms get tired more quickly and my knees start to hurt. Before I could go for longer, but now I can’t be in the same position for very long.”* (IDI8)

#### I'm having a good day today

The participants describe the importance of seizing the good moments, and fully embracing sexual encounters or quality time as a couple. While these moments cannot be planned, they can still nourish and revitalise the relationship.

*“When you’re in pain, your morale is at its lowest. It’s rock bottom. You don’t even want to look at yourself. So, you have to cheer yourself up and say: let’s take advantage of the moments when I’m feeling good because I don’t know how I’ll be tomorrow. That’s why I can’t plan anything, because I never know how I’m going to be feeling the next day […]; but on the days that I am feeling good I say, let’s take advantage of this moment now that I’m feeling better, and that’s what I do.”* (IDI7)

Disconnecting from stress, pain, and everyday problems, and engaging in activities in nature are useful ways to connect as a couple.

*“When we go somewhere just the two of us, we plan it […]. He knows what I like to do and takes me to places I like to go. On those days, we usually end up sleeping together. When you go to the countryside and get some fresh air, you disconnect from it all.”* (IDI6)

Planning a date can awaken desire in women with FMS. This can foster a predisposition to be with the partner and the heightened need for connection. Although their relationships require adaptation to pain, they actively seek solutions.

*“When we get to be alone and are relaxed, then yes, we’ll do it. Sometimes we go away for the weekend and I associate it (with sex) in one way or another, not with penetration but with masturbation. […] I still have desire and I can have orgasms. When I have sex, in one way or another, I always have an orgasm”* (IDI10)

The relationship is nurtured by routines that strengthen the couple’s bond. The result of these encounters is the activation of desire and sexual relations. Beyond pleasure, the study participants perceive that engaging in sexual relations has a positive effect on the body, mood, and symptoms of FMS. As one woman expresses it:

*“The next day I’m the happiest person in the world! The next day and during it, of course. Afterwards, your body is full of adrenaline and all the good feelings it gives you, you’re happy, relaxed, joyful. You’re over the moon. My husband looks at me and says: “your face even changes!”* (IDI10)

Having sexual relations can also be associated with decreased stress levels in participants, leading to improved sleep.

*“I think it allows you to release a lot of stress, if you’re in the mood and you’re not in pain. And even if you are in pain, you can rest up afterwards because there’s usually a pill to take […] I always take them to sleep, so when you combine both of those things you wake up feeling rested; it’s like when you have a muscle cramp and you get a massage and it loosens up.*” (IDI3)

#### When the desire killers arrive

The participants’ testimonies unanimously indicate a decline in sexual desire. Each woman attributes it to different factors, based on her experience, but they all agree that what kills desire is FMS. Sexual desire cannot be forced; it fades gradually. There is no desire for sexual relations due to pain, lubrication problems, stress, or lack of sleep.

*“Not sleeping, always saying that it hurts… how do you tell your partner that it hurts without it seeming like you’re making up an excuse not to do anything. When I’m tired, all I want to do is rest.”* (IDI9)

The participants view female desire through a distinctly psychological lens. As women, they understand that their sexual response is slower and requires more time to develop, a characteristic that is heightened in the context of FMS. This perception may be linked to sociocultural factors or the women's sexual education. As one participant says, they need to feel genuine interest and connection for their desire to be awakened.

*“What turns me off is that he’s very explicit. […] I need it to be calmer, more gradual… otherwise it’s impossible”* (IDI4)

The participants highlighted stress as the biggest enemy of desire, directly affecting sexual relations and the couple’s relationship.

*“If I’m stressed out, my sex drive drops to zero. When I’m under a lot of stress, I get really anxious and it's impossible for me to have sex of any kind. But it’s definitely physical, too […], My body is like a motorcycle going at 120 km/h, and it’s as if someone asked you to recite a poem while riding it. Look, my body just isn’t capable of anything else. There’s zero desire.”* (IDI5)

Pain is the other silent killer of desire; when it is present, desire immediately disappears. Women with FMS report that, although desire and the intention to have sex can happen at any time, their plans can be hindered by the onset of pain which causes frustration for both the woman and her partner.

*“When I’m in the mood and my husband is home, I’ll say to him: “later, when the kids leave, let’s take advantage of our alone time…”. But if I’m in pain later, when the poor thing comes in all excited, I have to tell him: “I'm really sorry but I’m not feeling up for it anymore.” This happens to me a lot and it’s hard to explain.”* (IDI8)

Some participants report that taking painkillers causes them to lose their sex drive. Aware of this, they often choose to learn to live with the pain rather than lose their sex drive and quality of life.

*“They completely kill my sex drive. I’ve tried several things but my body rejected them all. I didn’t feel like myself; I would get drowsy and feel like a zombie. When I was taking medication, I didn’t feel any sexual desire. As soon as I realised, I stopped taking it. I just started to just live with it and deal with the pain.”* (IDI3)

Another common side effect of treatments, such as antidepressants, is vaginal dryness. Lack of lubrication is linked to decreased libido and pain during sexual intercourse, leading to the need for lubricants to address the problem. As one woman explains:

*“I do think it has affected me because ever since all of this started and I was diagnosed with FMS, I’ve experienced vaginal dryness. Now I have to use lubricant, which I’d never used before, because with the increased dryness I have less desire. If I don’t use lubricant, it’s much more difficult for me”* (IDI7)

### Theme 2. Only your partner can understand the traffic light of desire

The partner is a key support system when dealing with FMS. For desire to be rekindled, women need affection, understanding, respect, and to be valued by their partners. They want to be “seen”, feel desired, recall shared memories, and reconnect with their partners from a place where pain is not the main focus. Understanding, patience, and communication are shared elements that can be interpreted by the partner to determine whether the red or green light of desire has been turned on.

#### If you’d look at me…

It is fundamental for the partner to be understanding of FMS in order to validate the women’s feelings. Without this empathy, the participants feel misunderstood and begin to lose the connection with their partner.

*“If it (FMS) were a more recognised condition, it wouldn’t be seen as something women make up. I think if they (partners) understood the condition a bit better, relationships would be different. […] If they knew it was something that is real and not just something made up in our heads, things would improve”* (IDI3)

When the partner truly understands the magnitude of FMS, its nuances, symptoms, and timing; women report that their bond and self-esteem are strengthened. It sometimes takes time, but it is the best way to move forward.

*“When he gets home and sees what I’m doing, he can sense how I feel. It’s not the same for him to see me cleaning or arguing with the kids as it is to see me lying on the sofa under a blanket. For him, that’s a red light; he’s not going to try to initiate or ask for sex.”* (IDI5)

When women feel more understood, they try to bring out the best in themselves to connect with their partners during the times that they can have intimate encounters.

*“I feel better now that he’s learned to understand me. He knows that the days when I say “I can’t,” means that I truly can’t. It’s brought us closer knowing that he respects me. When I say “not today” (sex), he accepts it. And that’s why I try to make it up to him whenever I can.”* (IDI7)

Understanding and empathy reduce stress and boost self-esteem, allowing participants to experience a rebirth of their womanhood. As one participant says, the pain fades, desire awakens, and they enjoy feeling attractive again.

*“It’s true what he (my partner) says. You need to step out of the roles of mother, sick person, or worker, and just be yourself. Feeling understood helps you to reconnect with yourself as a woman, to be yourself again, go out to dinner, look your best, and smell nice. Even if you’re hurting on the inside and taking pills (which don’t take the pain away), it hurts less because you’re not focused on the pain.”* (IDI5)

Patience is another key factor for a successful relationship. Understanding first, and patience second; this makes women feel valued, allows them to relax, and eliminates the need to struggle to be understood.

*“To those who don’t understand their wives, I would tell them to educate themselves and have a lot of patience; but there are some partners who just don’t get it. […] My husband says he needs to have a lot of patience with me, and it’s understandable. He sees that I’m unwell, he knows what’s going on, what hurts me, what my face looks like, when my mood changes.”* (IDI6)

Patience and understanding are vital; the woman is the priority. Sexual intimacy involves reconnecting through travel, visiting new places, and recalling the past.

*“You know what really makes me happy? Listening to songs from when we were dating; I get emotional because they remind me of when we were young and the spark we had. When you listen to those songs, you remember the place you were when you had your first kiss, which song you did what to. But you have to want it and let yourself go there, because if you don’t, you won’t feel it. When I look back on how we used to be and what we felt back then, the innocence and passion, I get sad thinking: and now what? But here we are, all these years later. My husband and I have grown to love each other in a different way.”* (IDI14)

#### When things don’t flow smoothly…

Oftentimes, marital responsibility leads some participants to experience their sexuality as a couple from a self-imposed obligation to have sex, even if they are not in the mood or are not feeling well.

*“His patience has limits too and obviously I feel bad because I know I can’t give him what we both need […] because he needs it, but so do I […] When we’re intimate, I feel proud of myself, like I’ve fulfilled my role as a woman. I’m relaxed and not worried that I’m not satisfying my partner as I should be […]. And I tell myself, despite everything, today’s been a good day because I was able to get through it all.”* (IDI7)

Other participants feel pressured by their partners. This, in part, makes them feel obligated to have sex without desire. This behaviour creates a rift between partners.

*“Imagine feeling obligated to do it… I’ve felt that many times. So, I’m like ok, come on, let’s just get it over with so that he’ll leave me alone. They want to and you’re expected to be willing, but you’re in pain and they don’t understand…”* (IDI12)

When sexual encounters take place but their body or mind is not ready, women agree that it is better to stop, try again another time, and wait until their physical condition is more conducive to sexual activity.

*“If we’re having sex and it starts to hurt, I tell him that I have to stop and he understands. But lots of times it bothers him, and I get it. He’s human too. I’ve always tried to deal with it by meeting him halfway.”* (IDI11)

*“Normally, when we’re going to have sex it’s because we both want to, right? But then you get into bed and start saying things like “Don’t touch me there! Don’t kiss me there! Don’t touch my hair! Ugh, I’m starting to get tired.” Then, the other person says: “Let’s stop, we’re not getting anywhere like this, neither of us are.” […] So, I’ll say: “Come on, let’s try again, let’s not end on a bad note”. But the same thing happens and you end up saying: “No, let’s stop because I’m getting in a bad mood, I’m getting mad at myself because I want to but I can’t”. I lose the desire and say: “That’s it. I’m done. We’ll try again tomorrow or another day, or whenever we feel like it again!” Maybe the following day it’s possible, but then not again for one or two weeks; and if I continue to feel unwell, I stop even trying. My partner notices and doesn’t initiate it either.”* (IDI6)

While many couples navigate these situations with patience and empathy, other participants do not report experiencing such understanding. In some cases, the situation leads to an increase in individual female desire or to fantasising about encounters with strangers to whom they would not have to justify their situation. As one woman explains:

*“When you’re alone, you sometimes think about and feel desire more intensely because at the end of the day, you understand yourself best. You know that you exist and that you’re still a woman. It’s difficult to deal with when you’re with someone who knows that you have something going on and doesn’t treat you with understanding (a partner), so I think about being with strangers, who don’t know what’s happening to me and won’t question me or look at me differently. (Your partner might think: “she’s already complaining, making up excuses”) […] Sexual desire with yourself is different; I know who I am, I understand my body, I know what I’m going through and what my limits are. It’s not the same with a partner, that’s why you fantasise about being with a stranger…”* (IDI3)

The majority of the participants live with a partner, and their experience of desire is primarily situated within this context. They distinguish between desire when alone and desire when with a partner, but find no difference between the two: “*No, I don’t feel desire when I’m alone, either*” (IDI8). For one participant who is single, if desire is absent, it is absent both when she is alone and when she is with someone else: “*I think it makes no difference whether it’s with another person or on your own; it depends on your mood and how you’re feeling in that moment.*” IDI2.

Another situation that disrupts the flow of the relationship is when pain and orgasm occur simultaneously. As one participant notes, orgasm and intense pain can coincide unpredictably.

*“Sometimes, before an FMS flare-up, orgasms are really painful. After I have one, I feel pain internally, it’s indescribable […] And of course, I’d say “It can’t be possible that an orgasm hurts. It can’t be!” I’d get so angry with myself. It’s true that sometimes they hurt and sometimes they don’t. When they don’t hurt, I always say: “Please let this last!” But I don’t have control over it. You never know if it’s going to hurt or not.”* (IDI8)

Faced with all of these situations which can be difficult for the participants to understand, they seek advice from multiple healthcare specialists. They search for answers that will improve their sex lives, but often encounter a barrier of lack of understanding, apathy, silence, and incomprehension.

*“We’ve brought it up at doctor’s appointments and it’s as if it’s irrelevant or it’s not concerning. It doesn’t matter where we are, even at the gynaecologist. It’s like it’s unrelated, they say: “no, don’t worry about that.” But I am worried. It’s part of my life. FMS changes your sex life a thousand times over.”* (IDI8)

#### FMS belongs to both of us

Recognising the problem, talking about it, and developing coping strategies together can improve a couple’s sex life. Communication between partners is a fundamental first step. It is essential for maintaining a connection as a couple and preserving intimacy, not only on a sexual level.

*“Always thinking you’re tired, exhausted, falling into depression and sex gets put on hold. It loses its importance. But you have to talk about it with your partner. There are lots of ways to understand each other. Sometimes they simply see your face and say, “Ok, it’s not happening today.” And that’s just how it is!”* (IDI3)

Lack of understanding is another key factor that significantly contributes to the loss of desire. Connecting with one’s partner may serve as a precursor to desire, while not feeling understood can completely inhibit it. As one participant stated:

*“The least helpful thing is when they ignore you, they don’t notice how you feel or understand what you’re going through, […] When they tell you that you’re exaggerating your symptoms… you stop thinking positively about everything, especially sex.”* (IDI15)

The partner must also take responsibility for the situation, acknowledging that the problem belongs to both of them, recognising that circumstances have changed, and accepting and adapting to the woman’s needs in regulating the intensity and frequency of sexual encounters. One woman expresses it clearly:

*“At first, it’s really difficult, but it gets better. Men don’t understand that you don’t feel sexual desire, or that even just being touched bothers you. They say: “How can this hurt you?” Because it does, it hurts! But at the end of the day, they are understanding, and of course, my husband had to start being more gentle.”* (IDI8)

All of these changes must be accepted and integrated by both the woman and her partner. Facing them requires effort based on self-knowledge and relational connection, prioritising these over sexual satisfaction.

*“It’s less frequent (sexual relations) because you have to mentally prepare yourself, knowing that you need more time to get there. But even if I’m not completely satisfied by the end of it, I’m happy with myself for having spent those intimate moments with him, even if I don’t reach orgasm.”* (IDI7)

Setting aside time is another way to connect as a couple, knowing there is a space where something can be felt beyond pain. Participants commented that adopting routines like going out for dinner or for walks creates intimacy, an exclusive space for the couple that fosters desire and sexual relations.

*“Every Saturday my husband makes me go out with him, even if I don’t feel like it. I’ll be waiting for him at home in pyjamas and say: “We’re already here, I bought dinner.”[…] But he insists. So, I get up from the sofa, take off my pyjamas, get dressed, put on my makeup, go out to eat, and talk about things other than the pain and pills… It changes your mood. When you get home, you’re in a completely different state of mind.”* (IDI5)

In any case, the woman’s partner can help her to relax, reduce stress, and incorporate play and affectionate touch that facilitate sexual intimacy. The key is not to give up or dwell on the past, but to adapt to the new situation.

*“Many people live their lives saying: I used to be, I used to do…” But you are not the person you used to be. You’re different. So, learn to live with who you are now.”* (IDI13)

## Discussion

The objective of this study was to describe and understand how women with FMS perceive their sexuality and the associated changes in desire. As in other studies, (Matarín-Jiménez et al., 2017), our results corroborate that the activation of sexual desire is severely compromised in women with FMS (Besiroglu & Dursun, 2019; Granero-Molina et al., 2023). For Merleau-Ponty, “we are a body” and perceive the world through a bodily schema. FMS transforms the lived body, while “desire killers” interfere with a body that is no longer recognised as a foundation of female sexuality. The hormonal changes can also reduce desire (Cuevas-Toro et al., 2025; Mutti et al., 2021), generating uncertainty in women and their partners. The participants describe an embodied experience marked by distress; their bodies no longer function as a source of desire or as a foundation of their femininity, generating uncertainty. We agree with Chesli et al. (2024) that low sexual self-esteem in women with FMS negatively affects the activation of desire, lowers the intensity of sexual encounters, and leads couples to develop coping mechanisms (Romero-Alcalá et al., 2019). Women experience distress because they have lost the bodily connection they once shared with their partner. Our participants exhibit low sexual self-esteem, which may be related to low sexual desire (Torres-Obregon et al., 2019). The anticipation of pain in FMS reshapes women's perceptual experience, shifting sexual encounters from the realm of pleasure to that of threat; thereby offering a phenomenological explanation for their loss of self-esteem. The participants stated that their desire fluctuates: decreasing with pain (de Carvalho et al., 2021; Rocamora-Pérez et al., 2025) and increasing with improved mood (Frérart et al., 2025). However, while sexual relations can improve symptoms, they are not always pleasurable (Matarín-Jiménez et al., 2017). Other research highlights the inverse correlation between FMS and sexual desire; “desire killers” include tiredness, depression, muscle stiffness, fatigue, stress, and pain (López-Rodríguez et al., 2019; Mutti et al., 2021); although other studies are inconclusive (Van Overmeire et al., 2022). Desire killers alter the female body schema causing the body to suffer and altering its perception of its own potential. This may explain why women with FMS feel less desired, experience a lack of desire, feel enclosed within themselves, and become distanced from their partners. Our results corroborate that the side effects of pharmacological treatments, such as poor lubrication (Leemans et al., 2025), generate discomfort, cause low arousal and desire, decrease the frequency of orgasms, and reduce overall sexual satisfaction (Deggerone et al., 2024; Van Overmeire et al., 2022).

This situation generates uncertainty and frustration, desire no longer governs women's sexual lives; rather, the timing of the FMS does. Our results do not indicate a shift in the participants' sexual identity; however, they recognise that they must live with (rather than fight against) FMS and that they need to change the way they experience their sexuality. According to our results, stress reduction, quality time, and satisfaction (Blumenstock et al., 2024) strengthen the couple's relationship. Equality and understanding are key for women (Wieczorek et al., 2022); a lack of support diminishes the activation of desire and sexual intimacy (Romero-Alcalá et al., 2019). The participants shared that arousing desire in an explicit way does not work; they need erotic-romantic stimulation with less sexual intensity. Most women did not feel their partners truly understood the severity of their symptoms, yet they needed that connection. They speak of their partners with admiration and appreciate their support which is explained by the fact that the majority of the women were married and in stable relationships. They long to feel seen and understood. Perceiving themselves as attractive women helps restore their identity, self-esteem, and sexual desire (Granero-Molina et al., 2016). The patience of their partner can help rekindle desire (Gore-Gorszewska, 2023), nostalgia of recalling shared moments can reactivate their connection, rushing extinguishes the desire they might initially feel. Sexuality is an intentional projection toward the other; our findings corroborate the female desire for a shared intercorporeality in which the gaze and empathy take precedence. In line with other studies (Newton-John et al., 2025), the partner plays a key role in the development of dyadic coping strategies for chronic pain. However, they may sometimes struggle to empathise with the women, leading to doubts and emotional misalignment (Pereira et al., 2025). Women who lack sexual desire may engage in sexual activity to satisfy their partners due to a self-imposed obligation stemming from feelings of guilt (Granero-Molina et al., 2023). Some studies (Santos-Iglesias et al., 2022) confirm that FMS alters the sequence of sexual activity, the partner loses the initiative and waits for the woman to give the “green light.” This results in a disruption of shared sexual intentionality, wherein the female body loses its spontaneity and submits to the other person's expectations.

Communication is key to avoid misunderstandings; for example, women fear being left for not meeting marital expectations, whereas men do not have these same thoughts (Romero-Alcalá et al., 2019). Many cannot fathom how their husbands endure the emotional rollercoaster of living with a woman who has fibromyalgia. Others have witnessed an evolution in them as they moved from a lack of understanding to offering support and companionship throughout the process. In agreement with Cuevas-Toro et al. (2025), women with FMS stated that sexual intercourse and orgasms can be painful. This leads to a decrease in sexual desire, frequency, and satisfaction (Granero-Molina et al., 2023; Karpuz et al., 2024). For Peixoto (2023), the loss of sexual satisfaction is linked to the activation of individual female desire, yet we did not observe this in our results. The participants' accounts point to a bodily experience marked by distress, yet an unaltered sexual identity; the essence of the woman as a sexual being remains intact, but FMS blocks its expression.

In general, women with FMS do not find answers from healthcare professionals to improve their sex lives (Granero-Molina et al., 2016). Altered body perception, combined with the need to continuously renegotiate bodily boundaries, highlights the importance of empathetic and individualised professional relationships (Cioeta et al., 2026). According to Bernardi et al. (2026), recommendations from clinical professionals can influence how patients construct meaning, as well as their behavioural responses and care expectations. Professional communication, validation, and acknowledgement of the bodily experience can influence how women with FMS interpret symptoms, relate to their bodies, and manage their sexual lives.

### Limitations

The results of this study are not generalisable to populations with different cultural perspectives on female sexuality. The wide age range of the participants may be a factor to consider when analysing and interpreting the results. The average age of the participants is high, suggesting an underrepresentation of young women of childbearing age, a group in which sexual desire is higher. This study does not collect data on sex education, marital status, relationship duration, or the presence of depressive disorders; factors that are negatively associated with sexual desire. Several eligible women declined to participate in the study due to the discomfort they felt discussing sexuality, which may introduce selection bias.

## Conclusions

FMS has a severe impact on women’s perceptions of sexuality and quality of life. Women perceive significant fluctuations in sexual desire which is linked to loss of self-esteem, low mood, and infrequent sexual encounters. While the loss of desire can be masked by age-related hormonal changes, pain, or stress; sleep problems or lubrication issues are also “desire killers.” The partner’s role is fundamental in mitigating the fluctuations in sexual desire in women with FMS. These women seek understanding, recognition of their femininity, closeness, and a willingness to establish erotic-romantic connections that go beyond the purely sexual. Patience, empathy, and communication are the best tools for nurturing intimacy in the relationship. Addressing female desire issues in FMS involves recognising a shared responsibility over time, fostering gradual understanding, adapting sexual practices, or incorporating new elements that stimulate desire and enhance the couple’s life. It is necessary that health professionals create opportunities to discuss sexuality proactively but sensitively; validate sexual concerns as a legitimate component of health and quality of life; consider the combined effects of pain, fatigue, sleep disturbance, medication, self-perception, and relational factors; avoid overly biomedical communication or provide access to appropriate multidisciplinary pathways where needed. These recommendations should nevertheless remain proportionate to the qualitative design and the specific cultural and demographic characteristics of the sample.

## Data Availability

http://hdl.handle.net/10835/21904.
